# Racial inequities and biopsychosocial indicators in older adults

**DOI:** 10.1590/1518-8345.5634.3514

**Published:** 2022-03-21

**Authors:** Alisson Fernandes Bolina, Nayara Gomes Nunes Oliveira, Paulo Henrique Fernandes dos Santos, Darlene Mara dos Santos Tavares

**Affiliations:** 1 Universidade de Brasília, Departamento de Enfermagem, Brasília, DF, Brasil.; 2 Universidade Federal do Triângulo Mineiro, Departamento de Enfermagem em Educação e Saúde Comunitária, Uberaba, MG, Brasil.; 3 Bolsista do Conselho Nacional de Desenvolvimento Científico e Tecnológico (CNPq) 1D, Brasil.

**Keywords:** Aged, Health of the Elderly, Health Status Disparity, Ethnic Group Distribution, Race Factors, Geriatric Nursing, Idoso, Saúde do Idoso, Desigualdade em Saúde, Distribuição por Etnia, Fatores Raciais, Enfermagem Geriátrica, Anciano, Salud del Anciano, Disparidades en el Estado de Salud, Distribución por Etnia, Factores Raciales, Enfermería Geriátrica

## Abstract

**Objective:**

to analyze the association of self-reported skin color/race with biopsychosocial indicators in older adults.

**Method:**

cross-sectional study conducted with a total of 941 older adults from a health micro-region in Brazil. Data were collected at home with instruments validated for the country. Descriptive analysis and binary, multinomial and linear logistic regression (p<0.05) were performed.

**Results:**

Most older adults were self-declared white color/race (63.8%). Black color/race was a protective factor for negative (OR=0.40) and regular (OR=0.44) self-rated health perception and for the indicative of depressive symptoms (OR=0.43); and it was associated with the highest social support score (β=3.60) and the lowest number of morbidities (β=-0.78).

**Conclusion:**

regardless of sociodemographic and economic characteristics, older adults of black color/race had the best outcomes of biopsychosocial indicators.

## Resumo

**Objetivo:**

analisar a associação da cor da pele/raça autorreferida com indicadores biopsicossociais de idosos.

**Método:**

estudo transversal conduzido com 941 idosos de uma comunidade na microrregião de saúde no Brasil. Os dados foram coletados no domicílio e com instrumentos validados no país. Procedeu-se à análise descritiva e regressão logística binária, multinomial e linear (*p*<0,05).

**Resultados:**

a autodeclaração de cor/raça branca predominou entre os idosos (63,8%). A cor/raça preta foi fator de proteção para a autoavaliação da saúde péssima/má (OR=0,40) e regular (OR=0,44) e para o indicativo de sintomas depressivos (OR=0,43); e associou-se ao maior escore de apoio social (β=3,60) e ao menor número de morbidades (β=-0,78).

**Conclusão:**

os achados denotam que, independentemente de características sociodemográficas e econômicas, os idosos da cor/raça preta apresentaram os melhores desfechos dos indicadores biopsicossociais.

## Resumen

**Objetivo:**

analizar la asociación del color de piel/raza autoinformado con indicadores biopsicosociales de personas mayores.

**Método:**

estudio transversal realizado con 941 adultos mayores de una comunidad en una microrregión de salud en Brasil. Los datos fueron recolectados en residencia y con instrumentos validados en el país. Se realizó análisis descriptivo y regresión logística binaria, multinomial y lineal (*p*<0,05).

**Resultados:**

la autodeclaración de color/raza blanca predominó entre los adultos mayores (63,8%). El color/raza negra fue un factor de protección para la autoevaluación de la salud mala (OR=0,40) y regular (OR=0,44) y para el indicativo de síntomas depresivos (OR=0,43); y se asoció con el puntaje más alto de apoyo social (β=3,60) y el menor número de morbilidades (β=-0,78).

**Conclusión:**

los hallazgos muestran que, independientemente de las características sociodemográficas y económicas, las personas mayores de color/raza negra tuvieron los mejores resultados de los indicadores biopsicosociales.

**Highlights:**

(1) Most older adults were self-reported of white color/race.

(2) Black color/race was a protective factor for negative self-rated health.

(3) Black color/race was a protective factor for the indication of depressive symptoms.

(4) Black color/race was associated with the highest social support score.

(5) Black color/race was associated with the lowest number of morbidities.

## Introduction

In Brazil, based on the 1991 demographic census, the Brazilian Institute of Geography and Statistics (IBGE) adopted the criterion of self-classification according to skin color/race into five categories: white, black, brown, indigenous and yellow^1^. The racial composition of the older adult population in the country has been changing over the years. In the year 2000, 61.7% of Brazilian older adults declared themselves white, 29.5% brown and 6.9% black^2^ and, in the year 2015, there was a decrease in the proportion of white color/race older adults (52.2 %) and an increase in browns (37.4%) and blacks (9.2%)^1^. However, the changes that have taken place in the country in the economic, political and health sphere have not yet mitigated the inequalities in the health conditions of racial groups of Brazilian older adults^3^ ^-^ ^4^.

Racial inequities in health result from inappropriate living habits and access to social and health resources^3^ ^-^ ^5^. In this context, color/race is seen as a marker of social position^4^ which reflects on the distinct distribution of risk, protection and health hazards that accumulate throughout life^3^. 

Despite evidence that health and social conditions differ among older adults according to color/race^3^ ^-^ ^4^ ^,^ ^6^ ^-^ ^8^, there is controversy about whether changes in biopsychosocial indicators are directly associated with racial issues^9^. These indicators are commonly used in the assessment of the health of older adults and involve sociodemographic and economic characteristics^3^ ^,^ ^5^ ^-^ ^8^, health conditions, such as the presence of frailty^10^, polymorbidity^3^ ^,^ ^5^ ^-^ ^8^, indicative of depressive symptoms^3^ ^,^ ^8^, functional incapacity^5^ ^-^ ^6^ and low physical performance^6^ ^-^ ^7^, in addition to self-assessment of health^3^ and social support networks^8^.

In national^3^ ^-^ ^4^ and international^6^ ^-^ ^8^ studies, it was observed that health and social conditions differed among white, brown and black older adults. However, in other studies it was found, regardless of sociodemographic and economic differences and the context in which individuals are inserted, that health inequities cannot be directly attributed to color/race^9^. In this perspective, we sought to investigate this theme, with the aim of expanding knowledge and subsidizing clinical practice in the care of older adults.

It should be noted that ethnic or racial health disparities in older adults have been widely studied in developed countries, especially in the United States of America (USA)^11^. Although Brazil is a nation with ethno-racial diversity, scientific knowledge about biopsychosocial indicators among older adults with a racial background is still incipient^3^ ^-^ ^4^, which makes it difficult to understand the influence of racial inequities at this stage of life. 

Thus, the objective was to analyze the association of self-reported skin color/race with the biopsychosocial indicators of older adults.

## Method

### Study design

Household, analytical and cross-sectional survey carried out in the urban area of a micro-health region in the state of Minas Gerais located in southwestern Brazil. This study was developed in accordance with the Checklist for Reporting Results of Internet E-Surveys guidelines and Strengthening the Reporting of Observational Studies in Epidemiology (STROBE) for cross-sectional studies^12^.

### Population and sample

The population consisted of individuals aged 60 years old or more living in the urban area. The multiple-stage cluster sampling technique was used to define the sample. For this purpose, the coefficient of determination R^2^ = 0.02 was considered in a multiple linear regression model with 12 predictors, with a level of significance or type I error of α = 0.05 and type II error of β = 0.2, resulting in an aprioristic statistical power of 80%. Using the Power Analysis and Sample Size® (PASS®), version 13, the values described above were introduced and a sample size of at least 798 older adults was obtained. A percentage of 20% was added to this sample value considering the possibility of sampling loss, totaling 956 the final number of interview attempts. 

The following inclusion criteria were defined: being 60 years old or older and living in the urban area of the health microregion of Minas Gerais, Brazil. Older adults with cognitive decline, assessed through the Mini Mental State Examination (MMSE) were excluded^13^; with severe stroke sequelae; self-reported, severe or unstable Parkinson’s disease; and/or with communication problems such as deafness, uncorrected by devices and severe speech disorders. A total of 956 older adults were recruited, of which 15 had cognitive decline. Thus, the final study sample consisted of 941 older adults. 

### Data collect

Data were collected at the older adults’ homes from March 2017 to June 2018 through direct interviews and physical performance tests using instruments validated for the Brazilian context, as described below. Ten interviewers from the health area were selected, who underwent training, qualification and approach to ethical research issues. It is noteworthy that the interviewers were trained by researchers, members of the Collective Health Research Group, and followed up until they were able to demonstrate the skills needed to apply the instruments used in this study.

At first, the interviewers performed the cognitive assessment of older adults, one of the exclusion criteria, through the application of the MMSE, considering the cutoff points: ≤ 13 for illiterate, ≤ 18 for low education (1 to 4 incomplete years) and middle (4 to 8 incomplete years) and ≤ 26 for high education (≥ 8 completed years)^13^. 

### Explanatory and adjustment variables

Sociodemographic and economic data were obtained through the application of a structured questionnaire, developed and widely used by the researchers of this study, which includes the following information: gender (male and female); age group, in years old (60 to 69, 70 to 79 and 80 or more) and age (numerical variable); marital status (never married, married, widowed and divorced/separated); level of education, in years of study (no education, 1 to 3, 4 to 7 years and 8 or more) and education, in years of study (numerical variable); and monthly individual income, in minimum wages (without income, up to 1, 1 to 3, 4 and more). 

### Independent variable - color/race 

The self-reported classification of skin color/race (white, black, brown and yellow) was used as defined in the country’s demographic census^1^. Data were obtained through the question: How do you classify your skin color/race?

### Dependent variables - biopsychosocial indicators

The frailty syndrome was assessed using the five components of the frailty phenotype^14^: 1. Unintentional weight loss: assessed by the question: “Last year, did you lose more than 4.5 kg unintentionally (that is, no diet or exercise)?”; 2. Decrease in muscle strength: verified based on handgrip strength (HGS) using a manual hydraulic dynamometer. Three measurements were obtained, presented in kilograms/force (kgf) with an interval of one minute between them, considering the mean value. The cutoff points adjusted for gender and body mass index (BMI) were adopted: men (BMI ≤ 24.0 and HGS ≤ 29.0; BMI 24.1 - 26.0 and HGS ≤ 30.0; BMI 26, 1 - 28.0 and HGS ≤ 30.0; BMI > 28.0 and HGS ≤ 32.0) and women (BMI ≤ 23.0 and HGS ≤ 17.0; BMI 23.1-26.0 and HGS ≤17.3; BMI 26.1 - 29.0 and HGS ≤ 18.0; BMI > 29.0 and HGS ≤ 21.0)^14^; 3. Self-report of exhaustion and/or fatigue was measured using two questions (items 7 and 20) of the Brazilian version of the Center for Epidemiologic Studies depression scale. Older adults with scores of two or three on any of the questions met the frailty criterion for this item^15^; 4. Slowness in walking speed: walking time (in seconds) was considered. The person walked a total distance of 8.6 m, with the initial 2 m and the final 2 m not being considered for the calculation of the time spent in gait. Three measurements were taken, considering the mean value. For this purpose, the Vollo® professional chronometer, model VL-1809, was used as standard, and the cutoff points adjusted by gender and height were considered, for men (Height ≥ 173 cm and Time ≥ 7 s; Height > 173 cm Time ≥ 6 s) and for women (Height ≥ 159 cm and Time ≥ 7 s; Height > 159 cm and Time ≥ 6 s)^14^; 5. Low level of physical activity: measured by weekly energy expenditure in Kcal and measured using the long version of the International Physical Activity Questionnaire (IPAQ), adapted for older adults^16^. The classification used for this component considered active those who spent 150 min or more of weekly physical activity; and inactive people who spent 0 to 149 min of weekly physical activity^17^. Older adults with three or more of the items described above are classified as frail, those with one or two items as pre-frail and those with all negative tests as robust or non-frail^14^. 

The number of self-reported morbidities and self-rated health was measured by applying the instrument developed by the study researchers. The self-rated health variable was classified into three categories: very positive/positive, regular and negative/very negative. The number of morbidities was considered a numerical variable.

The Basic Activities of Daily Living (BADL) were measured using the Katz Index, adapted to the Brazilian context and composed of six items that measure the individual’s performance in self-care activities^18^. For each item, there are three possible answers, the first and second denoting independence and the third one, dependence^18^. For Instrumental Activities of Daily Living (IADL), we used the Lawton & Brody Scale (1969), adapted in Brazil^19^. This scale is composed of nine items that have three answer alternatives for each question: independence, need for partial help and need for full help/cannot perform the activity. Based on these instruments, older adults were classified as independent or dependent for BADL and IADL.

To measure physical performance, we used the Brazilian version of the Short Physical Performance Battery (SPPB), consisting of the sum of the scores acquired in the tests of balance, gait speed and standing up from a chair five consecutive times and with a total score that varies from 0 (disability) to 12 (better performance), that is, the highest score represents a better physical performance^20^.

Depressive symptoms were assessed using the Abbreviated Geriatric Depression Scale, validated in Brazil, consisting of 15 questions and with a total score ranging from 0 to 15 points^21^. The total sum of points greater than 5 was considered indicative of depressive symptoms^21^.

To identify the network and social support, the Network and Social Support Scale was used, translated and validated in Brazil^22^. The social network was measured using two questions, including: “How many relatives do you feel comfortable with and can talk about almost anything?” and “How many friends do you feel comfortable with and can talk about almost anything?”. Social support is measured by the frequency with which the person has material support, that is, the provision of practical and material resources, such as help at work or financial assistance; positive social interaction/affective support that reflect the possibility of having someone to perform leisure activities and offer physical demonstrations of love and affection; and emotional/information support, which consists of the social network’s ability to meet individual needs in relation to emotional problems and the fact that it can count on people to advise, inform and guide^22^. The final score for each of the dimensions ranges from 20 to 100 points, and the higher the score, the better the level of social support^22^. 

Communication independence was assessed using the Communication Skills Functional Assessment Scale (ASHA-FACS)^23^ applied to the caregiver/family member and composed of four domains: Social Communication, related to social situations that require interaction with the speaker; Communication of Basic Needs, that is, the reaction to situations of need and emergency; Reading, Writing and Numerical Concepts, which consist of the ability of the person to take a message, identify food labels and/or fill in small forms; and the Daily Planning, which involves the notion of agenda to be fulfilled and appointments, use of the telephone and calendar^23^. The ASHA-FACS is graded as a seven-point scale, which assesses the performance of communication along the “continuum” of independence, in terms of levels of assistance and/or readiness necessary for communication^24^. In this graduation, seven means that the individual has a proper performance in the item, without the need for any assistance; six - needs minimal assistance for proper performance; five - minimal to moderate assistance; four - moderate assistance; three - moderate to maximum assistance; two - maximum assistance; and one - not capable of certain behavior, even with maximum assistance for it. At the end, the weighted average is calculated, reaching the average value of communication independence^23^.

### Data analysis

Data were processed with double independent typing and in electronic spreadsheets in the Excel® software, which were later compared, with the purpose of eliminating the possibility of typing errors. The final database was exported to the Statistical Package for the Social Sciences (SPSS®), version 22.0, for data analysis purposes.

Descriptive statistical analysis was performed by frequency distribution (absolute and percentage) for qualitative variables and by measures of dispersion and centrality (mean and standard deviation) for quantitative variables. To analyze the association of color/race with the biopsychosocial indicators of older adults in the community, linear regression, binary logistic regression or multinomial regression models were used, depending on the nature of the dependent variable. Subsequently, these models were adjusted for the variables: gender, age (years old), marital status, education (years of study) and individual monthly income. For all analyses, the significance level (α) of 5% and the tests considered significant when p ≤ α were adopted. 

### Ethical aspects

The project was approved on May 9, 2017 by the Research Ethics Committee with Human Beings, protocol N. 2.053.520. The older adults were presented with the objectives and the Informed Consent Form, and provided with pertinent information. After their consent and signature of the aforementioned Term, the interview was carried out following the precepts established by Resolution 466/12 of the Ministry of Health.

## Results

Of the total number of study participants (n=941), it was found that most of them self-declared themselves as white (63.8%), followed by brown (25.3%) and black (10.9%). 

As shown in Table 1, the highest percentages of white, brown and black older adults were female, aged between 70 and 79 years old, married, with 4 to 7 years of education and monthly individual income of up to one minimum wage.

**Table 1:** Distribution of sociodemographic and economic characteristics according to self-reported skin color/race of older adults living in the health microregion (n=941). Uberaba Health Microregion, MG, Brazil, 2017-2018

Variables	Color/race							
	White		Brown		Black		Total	
	n	%	n	%	n	%	n	%
**Gender**								
Female	411	68.5	157	66.0	61	59.2	629	66.8
Male	189	31.5	81	34.0	42	40.8	312	31.2
**Age group (years old)**								
60 to 69	216	36.0	96	40.3	39	37.9	351	37.3
70 to 79	242	40.3	98	41.2	50	48.5	390	41.5
80 or more	142	23.7	44	18.5	14	13.6	200	21.2
**Marital status**								
Never married	41	6.8	13	5.5	9	8.7	63	6.7
Married	260	43.3	103	43.3	41	39.8	404	42.9
Widower	240	40.0	92	38.7	37	35.9	369	39.2
Divorced	59	9.8	30	12.6	16	15.5	105	11.2
**Education (years)**								
None	76	12.7	60	25.2	31	30.1	167	17.8
1 to 3	137	22.8	56	23.5	26	25.2	219	23.3
4 to 7	229	38.2	94	39.5	36	35.0	359	38.1
8 and more	158	26.3	28	11.8	10	9.7	196	20.8
Individual monthly income^*^								
no income	32	5.3	13	5.5	6	5.8	51	5.4
up to 1 wage	296	49.3	131	55.0	59	57.3	486	51.6
1 to 3	226	37.7	88	37.0	34	33.0	348	37.0
4 and more	46	7.7	6	2.5	4	3.9	56	6.0

* Minimum wage in force in the data collection period: 2017 (BRL 937.00) and 2018 (BRL 954.00)

Table 2 shows the distribution of absolute and relative frequencies, mean and standard deviation of biopsychosocial indicators of older adults living in the health micro-region (MG) according to skin color/race self-reported.

**Table 2:** Distribution of biopsychosocial indicators of older adults living in the health microregion (n=941) according to self-reported skin color/race. Uberaba Health Microregion, MG, Brazil, 2017-2018

Biopsychosocial indicators	Color/race		
	Whiten*(%)^†^/mean(SD)^‡^	Brownn*(%)^†^/mean(SD)^‡^	Blackn*(%)^†^/mean(SD)^‡^
**Frailty condition**			
Frail	155 (25.8%)	48 (20.2%)	29 (28.2%)
Pre-frail	279 (46.5%)	119 (50.0%)	38 (36.9)
Not Frail	166 (27.7%)	71 (29.8%)	36 (35.0)
**Health self-assessment**			
Very negative/negative	97 (16.2%)	45 (18.9%)	13 (12.6%)
Regular	246 (41.0%)	87 (36.6%)	31 (30.1%)
Positive/great	257 (42.8%)	106 (44.5%)	59 (57.3%)
Number of	6.50 (± 3.38)	6.83 (± 3.46)	5.72 (± 3.30)
**BADL morbidities^§^**			
Dependent	42 (7.0%)	19 (8.0%)	6 (5.8%)
Independent	558 (93.0%)	219 (92.0%)	97 (94.2%)
**IADL^||^**			
Dependent	449 (74.8%)	177 (74.4%)	74 (71.8%)
Independent	151 (25.2%)	61 (25.6%)	29 (28.2%)
Physical performance	8.00 (± 3.24)	8.32 (± 3.06)	8.17 (± 3.27)
**Indicative of depressive symptoms**			
Yes	147 (24.5%)	63 (26.5%)	103 (13.6%)
No	453 (75.5%)	175 (73.5%)	89 (86.4%)
Communication independence	6.46 (± 0.78)	6.36 (± 0.74)	6.31 (± 0.78)
Social support	88.02 (± 16.88)	87.76 (± 17.39)	90.15 (± 14.64)
Social network	5.07 (± 4.70)	5.40 (± 5.07)	4.83 (± 4.48)

* n = Absolute frequency; ^†^% = Percentage frequency; ^‡^SD = Standard derivation; ^§^BADL = Basic activities of daily living; ^||^IADL = Instrumental activities of daily life

It was found that the black color/race was consolidated as a protective factor for negative self-rated health (OR: 0.40; CI: 0.20-0.78) and regular (OR: 0.44; CI: 0.27-0.72) and for the indicative of depressive symptoms (OR: 0.43; CI: 0.23-0.79) after adjustment for potential confounding variables. Furthermore, it was identified that the black color/race was associated with the highest social support score (β: 3.60: CI: 0.07-7.14) and the lowest number of morbidities (β: -0.78; CI: -1.47- -0.08) regardless of gender, age, marital status, education and individual monthly income (Table 3).

**Table 3:** Unadjusted and adjusted regression models of the association of self-reported skin color/race with biopsychosocial indicators of older adults living in the health microregion (n=941). Uberaba Health Microregion, MG, Brazil, 2017-2018

Biopsychosocial indicators	Unadjusted regression analysis		Adjusted regression analysis*	
	Color/race		Color/race	
	Brown β^†^/OR^‡^(CI)^§^	Black β^†^/OR^‡^(CI)^§^	Brown β^†^/OR^‡^(CI)^§^	Black β^†^/OR^‡^(CI)^§^
**Frailty condition**				
Frail	0.72 (0.47-1.11)	0.86 (0.51-1.47)	0.67 (0.42-1.07)	0.91 (0.50-1.65)
Pre-frail	0.99 (0.70-1.42)	0.63 (0.38-1.03)	0.96 (0.66-1.38)	0.61 (0.37-1.03)
Not Frail	1	1	1	1
**Health self-assessment**				
Very negative/negative	1.12 (0.74-1.77)	0.58 (0.31-1.11)	0.85 (0.54-1.32)	0.40 (0.20-0.78) ^||^
Regular	0.86 (0.62-1.20)	0.55 (0.34-0.88) ^||^	0.72 (0.51-1.02)	0.44 (0.27-0.72) ^||^
Positive/great	1	1	1	1
Number of	0.33 (-0.17-0.84)	-0.78 (-1.49- -0.07) ^||^	0.22 (-0.28-0.72)	-0.78 (-1.47- -0.08) ^||^
**BADL morbidities**				
Dependent	1.15 (0.66-2.03)	0.82 (0.34-1.99)	1.23 (0.68-2.22)	0.97 (0.39-2.40)
Independent	1	1	1	1
IADL**				
Dependent	0.98 (0.69-1.38)	0.86 (0.54-1.37)	0.91 (0.63-1.33)	0.85 (0.51-1.40)
Independent	1	1	1	1
Physical performance	0.31 (-0.17-0.79)	0.16 (-0.51-0.83)	0.26 (-0.21-0.72)	-0.01 (-0.65-0.64)
**Indicative of depressive symptoms**				
Yes	1.11 (0.79-1.56)	0.48 (0.27-0.88) ^||^	1.03 (0.71-1.45)	0.43 (0.23-0.79) ^||^
No	1	1	1	1
Communication independence	-0.09 (-0.21-0.02)	-0.15 (-0.31-0.01)	0.00 (-0.10-0.11)	-0.038 (-0.19-0.11)
Social support	-0.25 (-2.77-2.27)	2.13 (-1.38-5.64)	0.54 (-2.00-3.08)	3.60 (0.07-7.14) ^||^
Social network	0.33 (-0.39-1.05)	-0.24 (-1.24-0.76)	0.49 (-0.24-1.22)	-0.09 (-1.11-0.92)

Reference category: white color/race. ^*^Adjusted analysis for the variables: gender, age, marital status, education and income; ^†^β = Linear regression coefficient; ^‡^OR = Odds Ratio (Binary or multinomial logistic regression); ^§^CI = Confidence Interval; ^||^p<0,05; ^¶^BADL = Basic activities of daily living; ^**^IADL = Instrumental activities of daily living

## Discussion

This study explored the association of self-reported skin color/race with biopsychosocial indicators in a representative sample of community older adults in a micro-health region in Minas Gerais, Brazil. The findings showed that: a) most of them self-declared as being of white color/race, followed by brown and black; b) black color/race was consolidated as a protective factor for negative and regular self-rated health and for the indication of depressive symptoms, regardless of gender, age, marital status, education and income; c) black color/race was also associated with a higher social support score and a lower number of morbidities, even after adjustment. 

According to the National Household Sample Survey (NHSS), most of the general population in Brazil in 2018 declared themselves brown (46.5%), followed by white (43.1%), black (8, 3%) and yellow or indigenous (1.2%). However, when analyzing these data according to age group, it was found that, among Brazilian older adults, most self-reported as white (50.7%), followed by brown (39.2%) and black (8.8%)^25^, which corroborates the findings of this study and demonstrates a sample alignment in relation to the older adults’ population in the country.

The highest rate of self-declared white older adults is in line with the country’s mortality and life expectancy statistics, which show a higher proportion of early death among blacks and browns^3^. These data reinforce racial inequities observed in Brazilian society, causing many black people to not experience old age^26^, especially those in unfavorable living, health and socioeconomic conditions. 

As a result, according to a study carried out in the USA, in Pittsburgh and Memphis, older adults who declared themselves black had lower survival rates when compared to white ones^7^, which would result in the survival bias of those who are part of the most vulnerable racial groups^27^, that is, a more select sample of surviving black people. In line with this assumption, the study showed a lower percentage of black and brown older adults aged 80 years old and over compared to white ones. In order to minimize the confounding effect in this study, the association of self-reported skin color/race with biopsychosocial indicators was adjusted for the age variable.

In addition, another fact that drew attention was the higher percentage of brown and black older adults without education compared to white ones. It is noteworthy that health literacy can interfere with the self-perception of black people’s health^11^. Considering that education is one of the causes of health inequity among older adults^3^, the analyzes were also adjusted for this variable.

In the world literature, contradictory findings were identified regarding the association of color/race with the biopsychosocial indicators of older adults^11^ ^,^ ^28^. If on one hand studies found that the racial inequities observed in health status remained independently of socioeconomic and contextual diversities^11^, on the other hand, evidence showed that this variable partially explained the analyzed outcomes, with socioeconomic inequalities and/or the social context had a more relevant effect on the health of the older adults^28^ ^-^ ^29^.

Regardless of the direct effect of this variable or in conjunction with socioeconomic aspects, it is essential to consider color/race to understand the health inequities of the older adults population in Brazil^29^. As for the self-rated health indicators of this study, a similar result was evidenced in a survey conducted with 3594 older adults in the USA, which found that black people were more likely to have a positive self-rated health (excellent/very good/good) compared to Hispanics and Chinese (p=0.015)^30^. In South Africa, a study carried out with 3284 older adults also found that white and brown color/race participants had worse self-reported health status compared to black Africans^11^. It is noteworthy that South Africa is a developed country, with a great ethical-racial and cultural diversity and with a mostly black older adults population^11^.

In Brazilian literature, on the other hand, previous findings are divergent; while some showed that black older adults had worse self-rated health compared to the white ones^29^ ^,^ ^31^, another study found no association between these variables^3^. It is known that racial/ethnic and cultural differences may reflect in the self-reported subjective measures of older adults^27^ ^,^ ^32^ such as self-rated health. Therefore, this measure may not represent the same aspects between different racial/ethnic and cultural groups, which makes the differences in the findings between the aforementioned studies consistent. 

Some American scholars have promoted the theory that black individuals, due to material deprivation and/or racism throughout life, develop healthier adjustments to deal with adversities in relation to white ones^33^ ^-^ ^34^. Therefore, it is possible that the black color/race as a protective factor for negative health self-assessment in this study is related to this group’s greater coping capacity to deal with the challenges arising from senility and senescence, acquired in the past. 

Additionally, older adults who make up racial/ethical minorities, such as African-Americans, tend to maximize resources and optimize their well-being^35^, with a positive impact on mental health. This data is consistent with this study, as it verified that black color/race was shown to be a protective factor for the indication of depressive symptoms. A similar result was evidenced in a cohort study in Brazil, in which depression was less prevalent among black people compared to white ones (p=0.013)^3^. In a cohort study in the USA, it was observed that black older adults had higher levels of depressive symptoms compared to white ones. However, in a situation of higher chronic exposure to stress, those were less likely to report these symptoms compared to these^36^.

It is possible that black individuals who have experienced situations of segregation, racial discrimination and/or economic deprivation throughout their lives may have developed resilience, which, consequently, represents a protective factor against depression during old age^36^ ^-^ ^37^.

In this study, black color/race was associated with a higher social support score, which can be explained by the greater development of social cohesion attributes compared to the white population. This finding can also be understood as an adjustment or adaptive response to high exposure to stressors resulting from structural racism^33^ ^,^ ^37^.

Thus, it has been investigated whether social support influences black older adults’ health. A study developed with North American black men showed that social support, when assessed individually, was predictive of better self-rated health. However, when evaluated collectively with other psychosocial resources (e.g., optimism, sense of mastery and religiosity), it did not show significantly protective effects^38^. It is worth mentioning that the nurse plays an important role in caring for older adults in the community. Therefore, the social support network is one of the aspects that must be included in the nursing consultation^39^.

In addition, an association of black color/race with the lowest number of morbidities was identified, regardless of gender, age, marital status, education and income range, which should be carefully analyzed, as it differs from what has been shown in the literature. An investigation with groups of black and white older adults in Birmingham, Alabama, who had a diagnosis of diabetes mellitus, did not show significant differences in the prevalence of chronic diseases between the two racial groups, except for systemic arterial hypertension^40^.

In turn, a study that analyzed the relationship between color/race and health indicators of 18684 Brazilian older adults, included in the 2008 PNAD database, found a high prevalence of chronic diseases in those who declared themselves black compared to whites and browns^29^. Another investigation, based on data from the 2010 SABE study with a sample of 1345 people showed that among those with black color there was a higher prevalence of systemic arterial hypertension (83%; p=0.003), diabetes mellitus (40.80%; p =0.005) and stroke/cerebrovascular accident (18.7%; p<0.001), which have been identified as the main causes of mortality in black people in Brazil^3^. 

Race contrasts in the presence of chronic morbidities may be related to the fact that groups of older adults evaluated and distributed by race/color are unequally exposed to several risk factors that influence the adoption of healthy behaviors or that pose a risk to lifelong health, favoring racial differences in chronic disease estimates. Thus, it is possible that the person self-declared as black present a greater number of chronic morbidities as a result of the conditions being associated with the most vulnerable social groups^29^.

Considering the results of this research, related to the association of black color/race with the lowest number of morbidities, it is important to emphasize that black people have greater difficulty in accessing health services due to structural barriers, social and economic aspects, as well as the cultural, ethnic and racial prejudice, as demonstrated in a review study^41^. Therefore, these access limitations can lead to a lack of diagnosis for chronic conditions and, consequently, influence the results of research that obtain the number of morbidities through self-report.

It is noteworthy that the survival bias of black older adults may represent a potential limitation of this research and partially explain the findings by identifying that black color/race was associated with better outcomes related to biopsychosocial indicators after adjustment for gender, age, marital status, education and income range. Another possible limitation concerns the use of self-reported measurements that may be influenced by racial/ethnic differences.

The findings of this study reinforce the need to focus on nurses and other professionals who work in the context of primary care to ethnic-racial differences and their impact on the health parameters of the older adult’s population living in the health region under their responsibility through continuous monitoring of the health status of these individuals. This perspective favors comprehensive care and the provision of actions and services in an equitable manner, in line with the principles of the Unified Health System and primary care. It is also considered relevant to encourage the consolidation of registration of race/color in the documents of the Unified Health System, including the Health Information Systems^4^ enabling the monitoring of the health situation of the individuals in the country according to the racial cuts.

## Conclusion

When analyzing the association of self-reported skin color/race with the biopsychosocial indicators of older adults, it was found that black color/race was considered a protective factor for worse self-rated health and for the indicative of depressive symptoms and was related to the highest social support score and the lowest number of morbidities, regardless of sociodemographic and economic characteristics.
